# Keeping Public Health Clean: Food Policy Barriers and Opportunities in the Era of the Industrial Epidemics

**DOI:** 10.3934/publichealth.2016.2.228

**Published:** 2016-04-20

**Authors:** Martin O'Flaherty, Maria Guzman

**Affiliations:** 1 Department of Public Health and Policy; 2 Department of Public Health and Policy, University of Liverpool

**Keywords:** prevention of non communicable diseases, food policy, public health

## Abstract

Poor diet accounts for a larger burden of disability and death than tobacco, alcohol and physical inactivity combined.[1] The World Health Assembly has recognized this as a priority and has challenged member countries to reduce non-communicable disease (NCD) mortality by 25% by 2025 targeting their determinants.[2] Reaching these ambitious targets is possible, but it will require decisive action on diets and tobacco smoking if we want to make a difference.[1] Certainly diet can deliver these reductions rapidly, possibly in less than a decade, and particularly by reducing cardiovascular disease burden, still one of the most important cause of death globally. [3],[4]

But the impact of these diseases can be substantially lowered. Several natural experiments have shown the dramatic changes in mortality can be observed after changes of risk factors at population level, many attributable to changes in food intake [5]

## 1. Structural population level in changes delivers massive and rapid gains.

There are many natural experiments and policy interventions that massively change diets in a population. The first “hints” were observed in studies from Europe during the second world war, with coronary heart disease mortality rates in the UK associated to food rationing and after the infamous “hunger winters in the Netherlands and Norway.[6] In the 90s, and associated to the seismic social and economic changes that occurred the collapse of the Soviet Union. In Russia and other ex-soviet republics, these changes resulted in huge increases in mortality, most likely linked to increased alcohol consumption, social stress, tobacco smoking and raising uneployment.[7],[8] However in central Europe, the event led to dramatic and rapid declines in cardiovascular disease mortality, reversing decades longs increasing trends in mortality. [3],[9] In Poland, Slovakia and the Czech Republic, halving cardiovascular mortality trends over 5 years were largely explained by population wide changes in diet, including the overnight disappearance of subsidies for saturated fats that were mirrored by substantial increases in oils and fruit and vegetable intake.[9],[10] Cuba is another interesting example. During an extended period of deep economic crisis as a result of the dissolution of the Soviet Union in the 90s (“The Special Period”),the lack of economic subsidies resulted in severe shortages in the Cuban Economy. As a result, substantial declines in caloric intake accompanied by increases in physical activity, resulted in reductions in obesity and diabetes prevalence and crucially in CVD mortality.[11]

These natural experiments suggest that population level interventions can deliver gains of similar size and speed, but they can result in additional substantial economic and social gains.[12],[13] For example, mandatory salt reduction could result in 2 times bigger savings and 30% more life years gained than the voluntary scheme, and reduce by half the social gradient in coronary heart disease mortality.[14],[15] The same can be said for a total trans-fat ban.[16]

It is not surprising that structural modification of disease determinants at the population level is powerful. Public health can celebrate two centuries of successfully overcoming barriers to implement effective, structural policies for safe drinking water, clear air, safe motorcars, seatbelts, immunisations, smoke-free public spaces and minimal food contamination.[17] All of these are example of structural interventions, acting at the population level, and powerfully shaping the environment where people and societies live.

But this is a complex environment. Food production and distribution systems, education, marketing, regulation and cultural elements all interact in shaping population diets. However some of these “environments” are dominant; and particularly important is the role of “industrialization” on creating and sustaining epidemics.[18] The strong and continued trend on westernization of global diets, mainly driven by the concentrated industrialization of food production and the increasing consumption of ultra-processed food, is one of the key factors driving the NCD epidemic alongside population ageing.[19],[20]

Globally we have an increasing number of success stories around public health food policy. Historical exemplars in food policy such as Finland and Denmark are now being followed by countries such as South Africa & Argentina (salt), Mexico,Hungary & the UK (sugar) and Iceland and the USA (trans-fats). Europe has also an impressive array of food and nutrition policies aimed to improve health, but it still does not fully employ the powerful fiscal, regulatory and reformulation strategies that could result in substantial improvements in population diet.[21],[22] Specifically, whereas the policy landscape in Europe is well populated by health education workplace and school interventions and health promotion, powerful policies on product reformulation, regulation and price and fiscal policies tend to be less common, and implemented with varied intensity and spread.[22]

## 2. Food policy can benefit from replicating past Public Health successes

The majority of these successes represent the effective plod down a long policy path summarised by the mnemonic **SUPPORT**: **S**cientific evidence followed by **U**nderstanding by **P**rofessionals and gathering **P**ublic support, **O**vercoming Opposition from vested interests, usually overcome by **R**egulation and Taxation.[23]

Tobacco control has offer a wealth of evidence and experience in controlling this disease vector and it is a good example of the SUPPORT pathway. A comprehensive strategy will mirror the successes in Tobacco control, which was built on the “3As” model: addressing Affordability, Availability and Acceptability seems useful to tackle other determinants of NCDs. The use of a combination of fiscal measures, strict control of marketing and stringent regulation of point of sales,in a comprehensive package of measures covered in the WHO Framework Convention on Tobacco Control in have shown to be a powerful strategy to control the tobacco burden.[24] The UK based “Action on Sugar” campaign has championed the idea that “sugar is the new tobacco”.[25] The analogy is useful, since not only reminds about the powerful detrimental health effects, but also on the predictable response tactics of the industrial vector.[26] The Control of tobacco has been a difficult and continue to be a difficult challenge, and took several decades to achieve the current consensus, opposing powerful vested interest from the tobacco industry.[26] We shouldn't expect an easy path for our efforts to improve nutrition. But progress is now been made, and strategies to reduce affordability are now actively debated and implemented, focused on the obesity epidemic. [27] The emergence of sugar as a key driver of the obesity, diabetes and tooth decay have gained momentum in the past decade, prompting policy makers to take action.[28] A “soda tax” on sugar sweetened beverages has been implemented in Mexico, Hungary and Berkeley (USA). Initial evaluation of the tax in Mexico suggests that large effects on consumption are achievable, suggesting that these strategies work.[29] The intervention is being seriously considered in the UK,[28] with further actions to reduce availability and acceptability, by focusing action on marketing restrictions and health education, particularly targeted to children, and including powerful educational campaigns led by celebrities, like Jamie Oliver's “sugar rush”, are powerful tools to complement the fiscal measures.[30] Innovative policy frameworks like NOURISHING and INFORMAS propose specific actions and indicators to monitor and modify the food environment, food systems and behaviour change, and could help in further refining a global strategy to modify nutrition.

The above examples shared a focus on comprehensively controlling a set of determinants of diets . But crucially, It specifically targets the “industrial vectors” of the NCD epidemic with powerful structural interventions based on price and regulation, as discussed next.

## 3. Overcoming opposition to structural policies

The food industry has showed willingness to collaborate, and it could have a powerful role in improving population health. But the evidence of these voluntary agreements in terms of efficacy and safety is lacking.[31] Voluntary agreements that work usually have clear monitoring and targets, and substantial incentives or sanctions for non-compliance or missed targets, making them actually a “low intensity” mandatory intervention.[32] Thus, “weaker” implementations of these agreements are common and are actively embraced by the food industry in place of the more radical (and powerful) fiscal and regulatory interventions.

Powerful opposition from the food industry to these strategies is to be expected, as they usually restrict their room for manoeuvre and threaten profits. The “corporate disease vectors”[18] have considerable power, resources and experience in shaping environments in ways that maximize their profits, while not seriously and comprehensively considering its public health impacts. The tactics are similar to those used by the tobacco industry,[26] and particularly focused on blocking and delaying the more stringent fiscal strategies Some of the tactics used are targeted to deny the evidence of harm and thus effectively block evidence-based policy formulation.[33]

This set of tactics has been brilliantly summarized by Capewell et al in the acronym SLEAZE. They consist in allegation of Scientific conspiracies; the use of plausible arguments that are **L**ogically flawed, careful **S**election of the evidence to avoid the surfacing of conflicting facts, **Z**any arguments to drive attention away from the main issue, and blatantly buying **E**xperts to undermine good science or publish supportive evidence. Marion Nestlé's blog is providing some good evidence on the selective use and production of the evidence, and in its latest count, 75 out of 83 articles funded by food companies or trade associations provided results favouring the sponsors' interests, not very different from the known bias that the pharmaceutical industry has demonstrated.[34],[35] Another example of the use of these tactics is in contesting the emerging consensus of the major role of changes in the food system that produce an unprecedented availability of calories, modified by local socioeconomic, cultural and built environments[20], by supporting a major role of physical activity, as recently discussed in a blog article in the New York Times.[36]

This is not a new challenge for public health, and these obstacles were overcome in the past. Fighting tobacco is one of the greatest achievements of the past five decades, but at the expense of severe confrontations, as the struggle continues well into the beginning of the 21st century. However, the Framework Convention for Tobacco Control (FCTC) has proven to be recognition by the international community of the magnitude of the challenge and the commitment needed to overcome it. Learning again from anti-tobacco champions,[26] what we now need is to match the great achievement of health diplomacy like the FCTC, the Millenium goals,, World Health Assembly or the Revised International Health Regulations.[39] A way forward could be an international convention on healthy nutrition, that could result in economic, health and environmental benefits unlikely to be achieved by uncoordinated actions and lack of consensus. All of these policies can and should be implemented promptly and there is no lack of good practice examples with an expanding evidence base. This is becoming increasingly urgent, as there are reasons to believe that current free trade treaties being negotiated might pose a serious threat to the ability of states to leverage the powerful effect of regulation and legislation based interventions.[40]

When we think that we can achieve a 25% reduction in NCD burden by 2025 through dietary, alcohol and smoking policies, we are also implying that any deferment on its implementation will be costly. Every year we postpone the implementation of coordinated, effective, and evidence-based regulatory and market policies targeted at food, smoking and alcohol. This will not just cost us money, but it will also cost us thousands of lives and enormous environmental impacts.[41] The momentum for healthy diet and sustainable food systems is increasing at a fast pace alongside growing engagement from the public; what we lack is the political decision and commitment to implement them.

## References

[1] Kontis V, Mathers CD, Rehm J (2014). Contribution of six risk factors to achieving the 25×25 non-communicable disease mortality reduction target: A modelling study. Lancet.

[2] World Health Assembly (2013). Global action plan for the prevention and control of noncommunicable diseases.Geneva. http://apps.who.int/iris/bitstream/10665/94384/1/9789241506236_eng.pdf.

[3] Capewell S, O'Flaherty M Rapid mortality falls after risk-factor changes in populations. Lancet.

[4] (2015). Global, regional, and national incidence, prevalence, and years lived with disability for 301 acute and chronic diseases and injuries in 188 countries, 1990-2013: a systematic analysis for the Global Burden of Disease Study 2013. Lancet.

[5] Capewell S, O'Flaherty M (2011). Can dietary changes rapidly decrease cardiovascular mortality rates?. Eur Hear J.

[6] STROM A, JENSEN RA (1951). Mortality from circulatory diseases in Norway 1940-1945. Lancet (London, England).

[7] Notzon FC, Komarov YM, Ermakov SP, Sempos CT, Marks JS, Sempos E V (1998). Causes of declining life expectancy in Russia. JAMA.

[8] Ezzati M, Obermeyer Z, Tzoulaki I, Mayosi BM, Elliott P, Leon D a (2015). Contributions of risk factors and medical care to cardiovascular mortality trends. Nat Rev Cardiol.

[9] Zatonski WA, McMichael AJ, Powles JW (1998). Ecological study of reasons for sharp decline in mortality from ischaemic heart disease in Poland since 1991. BMJ.

[10] Bandosz P, O'Flaherty M, Drygas W (2012). Decline in mortality from coronary heart disease in Poland after socioeconomic transformation: modelling study. BMJ.

[11] Franco M, Bilal U, Orduñez P (2013). Population-wide weight loss and regain in relation to diabetes burden and cardiovascular mortality in Cuba 1980-2010: repeated cross sectional surveys and ecological comparison of secular trends. BMJ.

[12] Capewell S, Graham H (2010). Will cardiovascular disease prevention widen health inequalities?. PLoS Med.

[13] McGill R, Anwar E, Orton L (2015). Are interventions to promote healthy eating equally effective for all? Systematic review of socioeconomic inequalities in impact. BMC Public Health.

[14] Collins M, Mason H, O'Flaherty M, Guzman-Castillo M, Critchley J, Capewell S (2014). An economic evaluation of salt reduction policies to reduce coronary heart disease in England: a policy modeling study. Value Health.

[15] Gillespie DOS, Allen K, Guzman-Castillo M (2015). The Health Equity and Effectiveness of Policy Options to Reduce Dietary Salt Intake in England: Policy Forecast. PLoS One.

[16] Allen K, Pearson-Stuttard J, Hooton W, Diggle P, Capewell S, O'Flaherty M (2015). Potential of trans fats policies to reduce socioeconomic inequalities in mortality from coronary heart disease in England: cost effectiveness modelling study. BMJ.

[17] Smith R (2012). Learning from the abolitionists, the first social movement. BMJ.

[18] Jahiel RI, Babor TF (2007). Industrial epidemics, public health advocacy and the alcohol industry: lessons from other fields. Addiction.

[19] Monteiro CA (2011). The big issue is ultra-processing. There is no such thing as a healthy ultra-processed product. World Nutr.

[20] Swinburn BA, Sacks G, Hall KD (2011). The global obesity pandemic: shaped by global drivers and local environments. Lancet.

[21] Bromley H, Lloyd-Williams F, Orton L, O'Flaherty M, Capewell S (2014). Identifying the most effective and cost effective public health nutrition policy options for CVD prevention.

[22] Lloyd-Williams F, Bromley H, Orton L (2014). Smorgasbord or symphony? Assessing public health nutrition policies across 30 European countries using a novel framework. BMC Public Health.

[23] Capewell S (2014). Personal Communication.

[24] World Health Organization (2003). WHO Framework Convention on Tobacco Control.

[25] (2015). Action on Sugar. http://www.actiononsalt.org.uk/actiononsugar/.

[26] Brownell KD, Warner KE (2009). The perils of ignoring history: Big Tobacco played dirty and millions died. How similar is Big Food?. Milbank Q.

[27] England PH (2014). From evidence into action: opportunities to protect and improve the nation's health. https://www.gov.uk/government/publications/from-evidence-into-action-opportunities-to-protect-and-improve-the-nations-health.

[28] Public Health England (2015). Sugar Reduction: The evidence for Action.

[29] Instituto Nacional de Salud Publica (2015). Reducción en el consumo de bebidas con impuesto después de la implementación del impuesto en México. http://www.insp.mx/epppo/blog/3666-reduccion-consumo-bebidas.html.

[30] Sugar Rush | Jamie Oliver. http://www.jamieoliver.com/sugar-rush/#lA8MBiMVZdyt83td.97.

[31] Moodie R, Stuckler D, Monteiro C (2013). Profits and pandemics: prevention of harmful effects of tobacco, alcohol, and ultra-processed food and drink industries. Lancet (London, England).

[32] Bryden A, Petticrew M, Mays N, Eastmure E, Knai C (2013). Voluntary agreements between government and business - a scoping review of the literature with specific reference to the Public Health Responsibility Deal. Health Policy.

[33] McKee M, Diethelm P (2010). How the growth of denialism undermines public health. BMJ.

[34] Nestle M (2015). Food Politics » Another 5 industry-funded nutrition studies with results favorable to the sponsor. The score: 75:6. http://www.foodpolitics.com/2015/10/another-5-industry-funded-nutrition-studies-with-results-favorable-to-the-sponsor-the-score-766/.

[35] Naci H, Dias S, Ades AE (2014). Industry sponsorship bias in research findings: a network meta-analysis of LDL cholesterol reduction in randomised trials of statins. BMJ.

[36] O'Connor A (2015). Coca-Cola Funds Scientists Who Shift Blame for Obesity Away From Bad Diets - The New York Times. New York Times.

[37] Beaglehole R, Bonita R, Yach D, Mackay J, Reddy KS (2015). A tobacco-free world: a call to action to phase out the sale of tobacco products by 2040. Lancet.

[38] United Nations (2015). United Nations Millennium Development Goals.

[39] Katz R, Kornblet S, Arnold G, Lief E, Fischer JE (2011). Defining health diplomacy: changing demands in the era of globalization. Milbank Q.

[40] Weiss M (2015). Trading Health? UK Faculty of Public Health Policy Report on the Transatlantic Trade and Investment Partnership.

[41] Bailey R, Harper DR (2015). Reviewing Interventions for Healthy and Sustainable Diets.

